# Experiencing and Witnessing Patient Violence - an Occupational Risk for Outpatient Therapists?

**DOI:** 10.1007/s11126-019-09648-x

**Published:** 2019-05-27

**Authors:** Judith K. Daniels, Daniel Anadria

**Affiliations:** 1 Department of Clinical Psychology and Experimental Psychopathology, University of Groningen, Grote Kruisstraat 2, 9712 Groningen, TS Netherlands; 2 Psychologische Hochschule Berlin, Berlin, Germany

**Keywords:** Primary trauma, Assault, Physical attack, Treatment providers, Counsellors

## Abstract

Violence against therapists by their clients are a common occurrence across clinical settings and may have a lasting impact on the clinician’s professional and personal functioning. In spite of this, no study to date has looked at the frequency of trauma-induced symptoms in psychotherapists. Using a sample of *N* = 917 psychotherapists across Germany, Austria, and Switzerland, we analyzed the frequency and sequelae of patient attacks suffered or witnessed by therapists. More than half (51.3%) of the sample reported having been the victim or witness of patient attacks or threats of violence in their career. Among the affected therapists, 27.7% reported posttraumatic symptoms lasting longer than four weeks and 2.7% presented symptoms amounting to a full-PTSD diagnosis. Thus, while the frequency of attacks and trauma-induced symptoms were considerable, estimated PTSD rates were rather low. The findings suggest that practitioners should be conscious of client violence being an occupational risk and that it is advisable to have protective measures in place.

## Introduction

Mental health professionals experience incidents of patient violence or threats thereof in substantial numbers, with some estimates up to 60% [1–3]. The prevalence of such assaults has lately received increased attention of the scientific community in search of a better understanding of the phenomenon, its consequences, and prevention strategies [see review by 4].

Previous studies have reported on a wide range of incidents with patients, ranging from harassment to severe violence. For example, Hudson-Allez [5] reported that 21.6% of therapists received silent phone calls, 19.8% were waited for and approached outside of their workplace, and 19.8% felt physically intimidated by a client. Tryon [6] found that 81% of private practitioners experienced being either verbally harassed or physically attacked by a client during work. Almost half of the therapists sampled in Guy, Brown, and Poelstra’s [7] study had received verbal threats of physical violence from a client, and more than half of those threatened were attacked at some point with almost one in ten sustaining an injury. In general, the highest victimization estimates are found in studies conflating physical and verbal violence (e.g., 81% [6] and 61% [1]). Such outcome is to be expected knowing that incidents of verbal abuse often outnumber other forms of harassment [7, 8]. When not collapsed with verbal abuse, reports of physical violence towards therapists range from 14% [9] to 39% [7].

The work-setting seems to be an important determinant of the attack frequency. In general, inpatient hospital staff report a greater number of threats and assaults, as well as higher levels of fear, than therapists working in an outpatient setting [7, 9]. Tryon [6] showed that psychologists reported twice as many physical attacks when they worked in an inpatient setting as compared to an outpatient setting. Just under a third of all therapists sampled by Harris [10] reported having been physically attacked by a patient, with those working in an inpatient setting evidencing significantly higher incident rates. In a large sample of hospital psychiatrists, 72% reported having been threatened with and 65% having been exposed to physical violence [11]. These numbers are even higher for psychiatric nurses, with 72.2% reporting having experienced physical violence, arson, or threat thereof by a patient in the previous five years [12]. Younger, less formally educated, and less experienced staff particularly seem to be at higher risk of assault in inpatient settings [2].

Differences in patient composition might explain the differing frequency of attacks across work-settings. While most mental disorders are not accompanied by above-average aggressiveness, those that are commonly lead to hospitalization [12], at times in forensic settings [13]. Guy and colleagues reported that physical assaults on mental health professionals are carried out by predominantly male patients suffering from schizophrenia [7], while others also identified patients suffering from affective or substance use disorders as high risk groups [3, 14]. Other forms of harassment have predominantly been linked to patients with personality disorders, especially in response to the end of the therapeutic relationship [5].

Working with potentially aggressive patients, the mental health professionals must often make appraisals of their personal safety, which may differ by gender of both the therapist and the patient [15]. Guy, Brown and Poelstra [16] investigated the measures that therapists take to protect their personal safety. Among other strategies, therapists reported declining treatment to certain patients (50%), not sharing personal information with patients (41%), discussing safety with loved ones (30%), having measures in place in case something goes awry at the office (27%), and taking a self-defense course (4%). This may in part be due to the fact that violence prevention and management training is deemed insufficient by a significant number of therapists in training [17]. In fact, only 15% of therapists sampled by random selection from the APA therapist database in 1990 reported having undergone training in assault management [16].

Patient attacks can lead to an impairment of the therapist’s professional as well as personal functioning. A recent systematic review on sequelae of exposure to patient violence in nurses identified strong emotions such as fear, anxiety, and anger, but also shame and guilt [18]. In a study focusing on psychotherapists, just over half of the sample reported eating, concentration, and sleep disturbances due to fear of a client, especially those therapists who had survived a prior attack [19]. In spite of this, few studies assessed manifest symptoms of trauma-related disorders such as post-traumatic stress disorder (PTSD). While two studies carried out in Taiwan and in the United States found rather high prevalence rates of approximately 10% of full-blown PTSD in psychiatric staff [8, 20], a smaller study in Germany did not find a single subject meeting all diagnostic criteria [21]. In a later study, Richter and Berger [22] reported that 17% of mental hospital staff presented with PTSD seven weeks after being attacked by a patient, and 9% still fulfilled all diagnostic criteria two months later. In the United Kingdom, Wykes and Wittington [23] reported that 5% of the assaulted psychiatric nurses met diagnostic criteria. However, research on the consequences of patient violence is overall still scarce, especially in the outpatient setting, and to our knowledge no study has specifically investigated the resulting PTSD rates in psychotherapists.

### Aim of the Study

The aim of this study thus is to examine (1) how many psychotherapists were threatened with or exposed to physical violence at work, and (2) how many of them did in turn develop PTSD as a consequence of such experience.

## Methods

The study was conducted as an online study in German-speaking countries (i.e. Germany, Austria, and Switzerland). The link to the questionnaire was distributed widely, using repositories of contact details for outpatient therapists as well as internal emailing lists of several large-scale organizations offering counselling to trauma victims. In addition, the study was advertised in three professional journals tailored toward therapists. As a result, professionals with varying backgrounds as well as lay person counsellors participated in the study. The study went through the department’s ethical review process at the University of Bielefeld. Participants were presented with information about the study in the email and consented by completing the attached research package anonymously and sending it back without identifying information. We employed two different strategies to ensure that the measured symptoms did indeed stem from a direct, job-related trauma exposure – (1) the title of the study referred to work-related stress and the introduction made explicit reference to measuring exclusively symptoms related to experiences at work as well as the intention to differentiate between experiences in person and vicarious experiences; (2) we asked participants about traumatic events that they had experienced personally at the work place and measured PTSD symptom severity stemming from these experiences in line with the previous study by [22].

### Participants

In total, *N* = 1177 subjects participated in this study. After the exclusion of participants without a licensure for psychotherapy (*n* = 205, mostly lay counsellors, nurses, and social workers), 53 subjects with missing data as well as 2 psychotherapists who reported having worked for 0 years, a sample of *n* = 917 (605 females (66.03%), 308 males (33.6%), and 4 subjects identifying with neither of these two genders (0.4%)) working as licensed psychotherapists (either as a psychologist or a psychiatrist) remained. The following analyses refer to this sample. The participants’ age ranged from 25 to 69 (M = 47.17, SD = 8.39) and their total work experience varied between 1 and 47 years (M = 17.29, SD = 8.55). A subset of *n* = 846 participants reported how many of their patients had experienced traumatic events themselves. On average, 43.33% of the patients treated by the participants had been exposed to a traumatic event (range 0–100%).

### Instruments

The questionnaire battery was headed by the following introduction: “The goal of this study is to estimate the frequency with which work-related stress occurs. To this end, two forms of work-related stress will be differentiated: First, work-related stress which is the result of your personal experience including direct sensory input will be assessed. In the second part, work-related stress resulting from listening to experiences of our clients will be assessed” (all instructions translated from German by the authors). This was followed by items assessing socio-demographic data (age, gender, nationality, primary language, profession, work experience in years) and one item assessing the percentage of traumatized clients treated by the therapist. On average, it took participants 11.4 min to answer the whole questionnaire battery. Data on the effects of indirect exposure have been reported elsewhere [24].

### Direct Exposure to a Traumatic Event

Following DSM-IV diagnostic criteria for PTSD [25], both exposure to a traumatic event and emotional reaction to this event were recorded with two items each. The items were worded carefully so that they directly referred to a work-related experience (“Within your professional activities, have you ever experienced a physical assault personally or as a witness”, “Within your professional activities, have you ever experienced an acutely threatening situation, which could have led to injury or death”). Only subjects who reported both exposure to a traumatic event as well as having reacted with intense fear, helplessness, or horror were then asked to fill out the PTSD questionnaire.

### German Version of the Impact of Event Scale-Revised

The German version [26] of the Impact of Event Scale – Revised (IES-R) assesses the frequency of PTSD symptoms over the course of the last week. For this study, the introduction text was adapted to ensure that all participants report exclusively symptoms stemming from work-related trauma exposure. Participants rated 22 items referring to manifest PTSD symptoms on a 4-point Likert scale (‘not at all’ to ‘often’). While higher IES-R sum scores indicate stronger symptom severity, a formula for the tentative diagnosis of PTSD has been provided by the authors. To this end, the frequency ratings were re-coded to non-equidistant, uneven numbers (0 to 5) and subscale scores were computed for intrusions, avoidance, and hyperarousal. In the current sample, the questionnaire evidenced very high internal consistency (Cronbach’s Alpha = .924).

### Duration of Symptoms

The questionnaire was followed by an additional ordinal-scale item assessing the duration of the symptoms (answer options: “I did not experience any of the reactions described above”, “Reactions subsided within the first 4 weeks”, “Reactions lasted between 4 and 12 weeks”, “Reactions lasted between 3 and 6 months”,” Reactions lasted longer than 6 months”, “Reactions are currently still present”).

### Data Analysis

All analyses were carried out with SPSS 22. Chi Squared tests and t-tests were computed to compare exposed and non-exposed participants regarding their age, gender, work experience, and client composition. Bivariate correlations were explored between age, work experience, percentage of traumatized clients, and PTSD symptom using Pearson correlations. T-tests were conducted for group comparisons between exposed and unexposed participants. All statistical tests were run with a two-sided significance threshold of *p* < .05.

## Results

### Personal Exposure to Potentially Traumatic Events at Work

In total, *n* = 915 therapists provided information regarding their direct exposure to potentially traumatic events within the scope of their work. Of these, *n* = 368 (40.2%) reported having experienced physical assault either as the primary victim or a witness. With regard to situations with imminent threat of potential injury or death, *n* = 346 (37.8%) reported having experienced this. In total, *n* = 470 therapists (51.3%) reported having experienced at least one of these two events. Proportionally, significantly more male (62.3%, *n* = 192) than female (46.0%, *n* = 278) therapists reported having been exposed to physical assault or imminent threat (Chi2 = 21.943, *p* < .001).

Therapists exposed to physical violence or a situation carrying a high risk of physical injury were on average not older (M = 47.36, SD = 8.03; t(1, 889) = −.716, *p* = .474), but had more work experience (M = 18.09, SD = 8.15; t(1, 888) = −2.88, *p* = .004) than un-exposed therapists (age: M = 46.96, SD = 8.76; work experience: M = 16.45, SD = 8.89). Exposed therapists also reported treating a higher percentage of traumatized clients (M = 45.77%, SD = 27.78%) than their non-exposed colleagues (M = 40.7%, SD = 27.95%; t(1, 844) = −2.65, *p* = .008).

### PTSD Symptom Development

Of all exposed therapists, *n* = 271 (59.0%) reported either helplessness (*n* = 199, 43.4%) and/or fear (*n* = 197, 43.9%) and were thus asked to fill in the IES-R (*n* = 11 therapists (2.3%) did not answer these follow up questions). Of the n = 271 therapists invited to fill in the IES-R, *n* = 255 subjects did provide complete data. Following the potentially traumatic event at work, participants developed on average very low levels of PTSD symptoms as measured with the IES-R (sum score M = 22.29, SD = 17.91; intrusion subscale M = 8.21, SD = 6.80; avoidance subscale M = 6.68, SD = 6.49; hyperarousal subscale M = 7.41, SD = 6.98). According to the cut-off score [26], *n* = 7 therapists (2.7%) would have likely qualified for a PTSD diagnosis. The symptoms most often endorsed referred to heightened watchfulness (M = 2.37, SD = 1.06), emotional reactions to reminders (M = 2.24, SD = .923), and intrusions in the form of involuntary thoughts (M = 2.13, SD = .952).

### Symptom Duration

Of the *n* = 255 subjects with valid data for the IES-R, *n* = 246 provided information on the overall duration of the PTSD symptoms. The majority of participants reported symptoms having lasted less than four weeks (*n* = 133, 54.1%) or between one and three months (*n* = 39, 15.9%). However, *n* = 14 (5.7%) reported that the symptoms lasted between three and six months and *n* = 15 (6.1%) stated that they lasted longer than six months. Thirteen participants (5.3%) reported that the symptoms were still ongoing at the time of the inquiry.

## Discussion

The goal of this study was two-fold – to estimate how many psychotherapists are exposed to physical violence at work and to approximate how many of those subsequently develop PTSD.

The current results indicate that just over half of all therapists were exposed at least once to physical violence or a situation carrying a high risk of physical injury at their workplace. Of those experiencing intense helplessness or fear during the situation, just under 3 % reported subsequent symptom levels indicative of full-blown PTSD. While trauma-induced symptoms were likely to subside within the first month for approximately half the population, a sizeable portion was at risk of being impacted by the symptoms for more than three months.

### Physical Violence Estimates

Estimates of prevalence of client physical violence vary significantly across studies depending on their definition of violence [1, 4]. Studies not collapsing physical with verbal violence previously reported prevalence estimates for physical violence towards therapists ranging from 14% [9] to 39% [7]. The current study assessed work-related traumatic assaults where the therapist was either the primary victim or a witness. Thus, our finding that 40.2% of therapists have been exposed to physical violence at work is at the upper bound of the previous estimates, likely due to the inclusion of witnessing violence as a criterion. This was done in light of the fact that witnessing a physical assault is included in the DSM-IV definition of traumatic events [25]. Exposure to an acutely threatening situation was reported by 37.8% of the sample, bringing the total percentage of psychotherapists affected by at least one of the two to 51.3%. The finding that more than half of the participants reported at least one form of traumatic exposure clearly indicates that physical violence against psychotherapists is an occupational risk.

Interestingly, psychotherapists who reported having been assaulted by patients also reported treating a higher percentage of traumatized clients. While this likely reflects an effect of the high irritability and emotional dysregulation associated with traumatization, it could, in theory, also be a result of client selection on the side of the therapists. Future studies should aim to elucidate the processes underlying this association.

### Victim Characteristics

In our study, male therapists were more likely to be attacked than their female counterparts. While a similar pattern has been found in the previous research [7, 9, also see the review by 15, 27], little is known about the underlying mechanism of this difference. Levy and Hartocollis [27] hypothesized that the differing victimization rates in nursing staff may be linked to the self-fulfilling prophecy of aggressive patients expecting harsher and more police-like confrontation from male staff members. However, it remains unclear whether this is the case, and if so, whether the same mechanism would apply to psychotherapists (for a discussion on gender and risk of assault in psychiatric staff see [15].

Previous studies also found that inexperienced therapists were more likely to be assaulted [9], especially while still being in training [7]. In our study, the victimization rates were significantly higher for those with more work-experience, which was expected as we assessed lifetime prevalence rather than point prevalence.

### PTSD Symptoms

Several studies investigating PTSD rates in assaulted inpatient staff found clinical levels of posttraumatic symptoms in 5% [23] to 10% [8, 20, 22] of their samples [also see 28]. However, as these studies grouped together various mental health professionals who typically work in psychiatric wards, they may not be useful to estimate the prevalence of trauma-induced symptoms in psychotherapists who often work in outpatient settings. While many studies report descriptions of adverse sequelae of work-related harassment in therapists [5, 29], to our knowledge this may in fact be the first study to report specific trauma symptoms in this population. Using the recommended cut-off score to estimate PTSD prevalence based on self-report data, 2.7% of the exposed therapists would likely have received a PTSD diagnosis. This estimate being lower than the estimates for psychiatric staff (Caldwell, 1992; Chen, Hwu, Kung, Chiu, & Wang, 2008) can likely be explained by a higher tendency of psychological practicioners to work in outpatient settings where the rates of physical violence seem to be lower than in psychiatric facilities [7, 9]. While only 2.7% of the exposed therapists likely fulfilled all diagnostic criteria for PTSD, many additional practitioners reported experiencing some symptoms such as heightened watchfulness, emotional reactions to reminders, and involuntary intrusive thoughts. Even without meeting all diagnostic criteria for PTSD, these symptoms might impair their job performance and satisfaction [28].

### Limitations

While a strength of the study lies in its rather large sample size, it remains unknow to what extent the sample can be considered representative. As we used several recruitment channels and cannot ascertain via which channel our participants received the link, we cannot determine whether a recruitment bias might have impacted our study results. We also have no information about how many subjects received information about the study but opted not to participate. One might hypothesize that therapists who experienced physical violence on part of their patients may have been more interested in the study topic and might thus be over-represented. On the other hand, avoidance is a key ingredient of posttraumatic reactions and therapists suffering from posttraumatic symptoms might have opted not to participate in this particular study and would thus be underrepresented. In addition, the retrospective design might limit the validity of the reported symptom frequencies due to memory bias.

We opted to use the PTSD diagnostic criteria as defined by the DSM-IV that was valid at the time of assessment and thus employed the A2 inclusion criterion (referring to feelings of fear, horror or helplessness experienced during the traumatic event; [25]. In the meantime, this criterion has been abandoned as other peritraumatic emotional reactions such as shame or disgust are also related to PTSD. As we only presented the IES-R to those therapists who had fulfilled both DSM-IV A criteria for PTSD, the effect of the criteria change cannot be tested in our sample.

## Conclusion

In summary, our study found that more than half of the therapists have been exposed to physical violence or threat thereof at work. This was the case for more than 60% of male and 46% of female therapists. Although the overall PTSD rates were estimated to fall rather low, just under 3 %, in more than one in ten therapists the trauma-induced symptoms lasted for longer than three months, and more than 5 % reported having ongoing symptoms at the time of the inquiry. Client violence thus needs to be considered an occupational risk for clinical practitioners and clinicians should be made aware of the risk and be advised to have personal protection measures in place.
